# Longitudinal evaluation of glomerular filtration rate across adolescence and early adulthood in youth with type 1 diabetes: associations with clinical and social risk factors

**DOI:** 10.3389/fendo.2026.1888628

**Published:** 2026-08-26

**Authors:** Antoine B. M. Clarke, Jacqueline R. Curtis, Yesmino T. Elia, Paul Benitez-Aguirre, Fergus J. Cameron, Scott T. Chiesa, Cheril Clarson, Jennifer J. Couper, Maria E. Craig, R. Neil Dalton, Elizabeth A. Davis, Sally M. Marshall, David Z. I. Cherney, M. Loredana Marcovecchio, Farid H. Mahmud

**Affiliations:** 1 Division of Endocrinology, Department of Pediatrics, The Hospital for Sick Children, Toronto, ON, Canada; 2 Institute of Medical Science, University of Toronto, Toronto, ON, Canada; 3 Institute of Endocrinology and Diabetes, The Children’s Hospital at Westmead, University of Sydney, Westmead, NSW, Australia; 4 Department of Paediatrics, University of Melbourne, Parkville, VIC, Australia; 5 Institute of Cardiovascular Science, University College London, London, United Kingdom; 6 Children’s Hospital, London Health Sciences Center, London, ON, Canada; 7 Diabetes and Endocrinology Department, Women’s and Children’s Hospital, North Adelaide, SA, Australia; 8 Discipline of Paediatrics & Child Health, School of Clinical Medicine, University of New South Wales, Sydney, NSW, Australia; 9 Evelina London Children’s Hospital, Guy’s and St Thomas’ National Health Service Foundation Trust, London, United Kingdom; 10 Department of Endocrinology and Diabetes, Perth Children’s Hospital, Nedlands, WA, Australia; 11 Paediatrics Division, University of Western Australia, Crawley, WA, Australia; 12 Diabetes Research Group, Translational and Clinical Research Institute, Faculty of Clinical Medical Sciences, Newcastle University, Newcastle upon Tyne, United Kingdom; 13 Division of Nephrology, University Health Network and University of Toronto, Toronto, ON, Canada; 14 Department of Paediatrics, University of Cambridge, Cambridge, United Kingdom

**Keywords:** estimated GFR, kidney function, longitudinal, pediatric, transition to adult care

## Abstract

**Background:**

The continuous evaluation of GFR among youth with T1D has traditionally been difficult during the transition to adult care given distinct pediatric & adult estimated GFR (eGFR) formulas recommended by clinical practice guidelines. We aimed to assess longitudinal trends in kidney function in type 1 diabetes from adolescence to early adulthood using updated eGFR formulae and to examine associations between eGFR and clinical as well as social factors over time in this population.

**Methods:**

Clinical factors including age, sex, diabetes duration, insulin therapy, BMI and HbA1c in addition to social factors including ethnicity & material deprivation (MD) were collected alongside creatinine (Cr) and cystatin c (CysC) from Canadian participants in the AdDIT & CanSOLVE CKD-SPOR studies (2009-2023). Estimated glomerular filtration rates (eGFR) derived using updated Chronic Kidney Disease in Children under 25 (CKiD U25) Cr, CysC & combined Cr-CysC formulas were evaluated longitudinally using linear mixed effects regression, as were urine albumin-creatinine ratios (uACR).

**Results:**

Youth with type 1 diabetes ages 10 to <25 years were evaluated (N = 137) at a median 5 timepoints (IQR: 4-6) over 9.8 ± 1.3 years. As age increased, eGFR_Cr_ increased mildly (β:+1.0 ml/min/1.73m^2^/year; P<0.0001), whereas eGFR_CysC_ decreased until the age of 18 (β:-3.3 ml/min/1.73m^2^/year; P<0.0001) and stabilized thereafter (β: +0.1 ml/min/1.73m^2^/year; P = 0.433). These trends nullified one another across the age range using the combined eGFR_Cr-CysC_, which remained stable (β:-0.1 ml/min/1.73m^2^/year) from ages 10–25 years (P = 0.452). Sex, BMI, insulin therapy, HbA1c and MD were associated with eGFR_Cr-CysC_ over time with a large difference of +6.5 ml/min/1.73m^2^ seen in high relative to low MD groups (P = 0.0025). Log-transformed uACR was negatively associated with male sex (P = 0.017) and baseline diabetes duration (P = 0.049) yet positively associated with HbA1c (P = 0.021).

**Conclusions:**

Different eGFR trends were observed using Cr- vs. CysC-derived estimates in youth with diabetes using updated eGFR formulae. Elevations in eGFR, alongside alterations in markers of albuminuria, were associated with increased glycemia and marginalization, providing insights into key clinical and social risk factors for kidney disease monitoring in this population. (NCT01581476).

## 1 Introduction

Diabetic kidney disease (DKD) remains a significant complication of type 1 diabetes (1). Clinical practice guidelines recommend the evaluation of albuminuria alongside the routine monitoring of glomerular filtration rate (GFR) across the lifespan to identify those at greatest risk for early intervention aimed at mitigating long-term complications (1, 2).

Among youth with type 1 diabetes, the evaluation of DKD risk is limited by GFR estimating equations that do not allow for an accurate and continuous assessment of GFR trajectories across adolescence into early adulthood (1, 3–5). Commonly used pediatric-and adult-based GFR estimation formulas, such as the Chronic Kidney Disease in Children (CKiD Schwartz 2009) and adult Chronic Kidney Disease Epidemiology Collaboration (CKD-EPI) are not compatible and yield different results due to mathematical rather than clinical factors (3, 6, 7). Practically, these variations in estimated GFR during the transition from pediatric to adult care may complicate clinical decision-making related to changes in GFR over time (7, 8). Updated CKiD U25 formulas, that extend the upper age limit to 25 years (6), allow clinicians and researchers to monitor kidney function from adolescence into young adulthood, offering an opportunity to assess longitudinal trends in relation to clinical and social determinants of kidney disease in this population during this important transitional phase (9, 10).

Clinical characteristics including suboptimal glycemic control, obesity, diabetes duration and hypertension have been shown to be considerable risk factors for the progression of kidney disease in youth and adults with type 1 diabetes (11–13). While these traditional clinical factors are well documented in the diabetes literature, the impact of social factors is often overlooked. Given the significance of recognized social determinants of health on markers of renal function (11, 14) and DKD (15, 16), it is essential to accurately evaluate eGFR in youth with type 1 diabetes in a way that more broadly encompasses traditional and non-traditional risk factors. Social risk factors that encompass socioeconomic status (SES) and ethnicity are known drivers of chronic disease progression, making the evaluation of the intersection of social determinants with renal function important for potential risk stratification (17–20).

The aim of this study was to assess longitudinal trends in kidney function among youth with type 1 diabetes from adolescence into young adulthood. Using data from Canadian participants in the Adolescent Diabetes Cardiorenal Intervention Trial (AdDIT; NCT01581476) and its extension observational study spanning serial assessments over 10 years, this analysis aimed to evaluate trends in creatinine-based (Cr) and cystatin c (CysC) and combined Cr-CysC CKiD U25 eGFR over time, and to assess longitudinal associations between eGFR and markers of clinical heterogeneity including clinical and social factors.

## 2 Materials and methods

### 2.1 Study population & design

This retrospective, longitudinal cohort study evaluated N=137 Canadian participants recruited as part of the Adolescent Type 1 Diabetes Cardio-Renal Intervention Trial (AdDIT; 2009-2017) (21) and subsequently followed as part of the Canadians Seeking Solutions and Innovations to Overcome Chronic Kidney Disease- Strategy for Patient-Oriented Research (CanSOLVE CKD-SPOR; 2019-2023) follow-up study (22, 23). These studies were approved by the Hospital for Sick Children's Research Ethics Board (REB Nos: 1000012240 and 1000055749, respectively). The study protocol was conducted in accordance with the tenets of the Declaration of Helsinki and all participants provided written informed consent.

Study data including age, sex, diabetes duration, BMI, insulin therapy, self-reported ethnicity, Hemoglobin A1c and postal codes were extracted at 6 timepoints representing the baseline, midpoint, final study visits of both the AdDIT and CanSOLVE CKD-SPOR follow-up studies. SES was approximated using the Material Deprivation (MD) dimension of the Ontario Marginalization Index (ON-Marg) derived from 6-digit postal codes linked to dissemination areas (DAs) using the StatsCan Postal Code Conversion File (PCCF) (24). Individuals from the 3 least deprived MD quintiles (Q1-Q3) and those from the 2 most deprived MD quintiles (Q4-Q5) were grouped and analyzed as ‘MD quintile groups’ rather than individual quintiles to increase the power of the overall analysis. This decision was based on evaluation of baseline eGFR levels across MD quintiles, which revealed a similar low eGFR levels among Q1-Q3 opposite to more elevated eGFR levels among Q4-Q5 (see **Supplementary Figure 1**).

### 2.2 Laboratory testing

Data derived from samples collected during in-person study visits were used for this evaluation. Serum creatinine levels were analyzed by the Department of Pediatric Laboratory Medicine (DPLM) at The Hospital for Sick Children (Toronto, Canada). Creatinine testing was completed using the Ortho Vitros enzymatic assay (Rochester, USA) derived from a Gas Chromatography Isotope Dilution Mass Spectrometry (GC/IDMS) method alongside National Institute of Standards and Technology (NIST) SRM^®^ 914 creatinine standard reference material. Cystatin c levels were tested at Toronto General Hospital (Toronto, Canada) using Abbott Architect c8000. HbA1c was analyzed by the DPLM at The Hospital for Sick Children (Toronto, Canada) using standard laboratory methods. Urine albumin and creatinine values used to calculate urinary albumin:creatinine ratio (uACR) were derived from 3 early morning urine samples using the methods described in Marcovecchio et al. (25). Multi-day ACR values were averaged and used to define moderately elevated albuminuria using a cutoff of ≥3 mg/mol (1).

### 2.3 Estimates of GFR

GFR was estimated using the combined CKiD U25_Cr-CysC_ eGFR formula, the primary outcome for this study (26). The combined eGFR _Cr-CysC_ represents the average of the age-and sex-adjusted CKiD U25_Cr_ & CKiD U25_CysC_ equations and was chosen as it has been shown to better approximate measured GFR when compared to either of the individual formulas (27, 28). CKiD U25_Cr_ eGFR was derived using the following equation: [eGFR_Cr_ = K x height(m)/Cr(mg/dL)], whereas the CKiD U25_CysC_ eGFR was calculated by: [eGFR_CysC_ = K x 1/CysC(mg/L)], where K is constant that varies by age group and sex (26). As the CKiD U25 formulas are validated in individuals up to age 25, observations collected in participants above the age of 25 years were excluded from the analysis. Hyperfiltration was defined using an eGFR cutoff of ≥135 ml/min/1.73m^2^, as a well-recognized consensus threshold for whole kidney hyperfiltration in youth with type 1 diabetes (29).

### 2.4 Statistical analysis

All statistical analyses were completed using R statistics v4.5.1 (www.r-project.org) in combination with the R Studio v2025.09.1 + 401 integrated development environment (www.posit.co). Descriptive statistics for continuous data are presented as means ± standard deviation, while categorical data are as frequency counts and percentages. Cross-sectional comparisons of continuous outcomes were evaluated using two-sample T tests or ANOVA, or Mann-Whitney and Kruskal-Wallis tests if non-parametric. Categorical outcomes were cross-sectionally evaluated using simple chi-squared and/or Fisher’s exact tests.

Longitudinal differences in eGFR were evaluated using linear mixed effects regression with random intercepts and slopes for subjects over time using restricted maximum likelihood. The measure of time for this model, age in years, was also included as a fixed effect alongside sex, baseline diabetes duration, insulin therapy, BMI, HbA1c, ethnicity and socioeconomic status, as measured by Material Deprivation quintile groups from the Ontario Marginalization Index (ON-Marg) (24). Two-way interactions between fixed effects and time (age) were tested. A piecewise linear model with a knot at age = 18 was used for CKiD U25_CysC_ eGFR to optimize model fit, resulting in 2 regression slopes for ages <18 and ≥18 years, respectively. Longitudinal differences in categorical outcomes such as hyperfiltration and moderately elevated albuminuria were analyzed using Generalized Estimating Equations with an AR1 correlation structure. GEE models included the same subset of fixed covariates used as part of the linear mixed effects regression. Models for hyperfiltration and moderately elevated albuminuria were re-run using thresholds of 126.8mL/min/1.73m^2^, representing population-based thresholds + 2 SDS above the mean from the US-based NHANES study (30), and a lower abnormal ACR threshold of ≥2 mg/mol as recommended by Diabetes Canada (31) as part of sensitivity analyses. Confidence was set at 95%CI with significant associations declared at P<0.05.

## 3 Results

### 3.1 Participant characteristics

A total of N = 137 participants involved in both the AdDIT and CanSOLVE CKD-SPOR follow-up studies were analyzed. At baseline, mean age was 13.9 ± 1.6 years, females represented 46.7% of the sample and the average duration of diabetes was 6.1 ± 3.3 years. The average duration of follow-up from baseline to last observation was 9.8 ± 1.3 years, with participants completing a median of 5 (IQR: 4-6) serum creatinine & cystatin c assessments over that time. Mean HbA1c at baseline was 66 ± 12 mmol/mol (8.2 ± 1.1%), BMI was 21.9 ± 4.2 kg/m^2^ and 62.0% of the cohort was on insulin pump therapy. Complete baseline characteristics are presented in **Table 1**.

**Table 1:** Baseline characteristics of study participants.

Baseline characteristics	Overall sample N = 137
Age, years	13.9 ± 1.6
Diabetes Duration, years	6.1 ± 3.3
Sex	
*Female*, n (%)	64 (46.7)
*Male*, n (%)	73 (53.3)
Body Mass Index, kg/m^2^	21.9 ± 4.2
SBP, mmHg	113.8 ± 10.0
DBP, mmHg	66.6 ± 7.9
Insulin therapy	
*CSII*, n (%)	85 (62.0)
*Injections*, n (%)	52 (38.0)
HbA1c, mmol/mol	66 ± 12
HbA1c, %	8.2 ± 1.1
Ethnic background	
*White*, n (%)	79 (57.7)
*South Asian*, n (%)	13 (9.5)
*Black*, n (%)	8 (5.8)
*Chinese*, n (%)	8 (5.8)
*First Nations*, n (%)	1 (0.7)
*Other*, n (%)	28 (20.4)
MD quintiles	
*Q1 (Least Deprived)*, n (%)	33 (24.1)
*Q2*, n (%)	42 (30.7)
*Q3*, n (%)	31 (22.6)
*Q4*, n (%)	19 (13.9)
*Q5 (Most Deprived)*, n (%)	12 (8.8)
MD quintile groups	
*Q1-Q3 (High SES)*, n (%)	106 (77.4)
*Q4-Q5 (Low SES)*, n (%)	31 (22.6)
eGFR_Cr_ (CKiD U25), ml/min/1.73m^2^	111.0 ± 16.2
eGFR_CysC_ (CKiD U25), ml/min/1.73m^2^	108.8 ± 18.8
eGFR_Cr-CysC_ (CKiD U25), ml/min/1.73m^2^	109.9 ± 14.4
uACR, mg/mmol	1.43 ± 1.88
Albuminuria (moderately elevated)	
<3 mg/mol, n (%)	121 (88.3)
≥3 mg/mol, n (%)	16 (11.7)

### 3.2 Longitudinal trends in eGFR using U25 Cr, U25 CysC and U25 Cr-CysC equations

A total of N = 646 creatinine readings and N = 612 cystatin C readings were used to derive eGFR values for this analysis. Mean eGFR_Cr_ was 111.0 ml/min/1.73m^2^, eGFR_CysC_ was 108.8 ml/min/1.73m^2^, and eGFR_Cr-CysC_ was 109.9 ml/min/1.73m^2^ at baseline with hyperfiltration (eGFR_Cr-CysC_ ≥ 135 mL/min/1.73m^2^) observed in n=8 (5.8%) of participants at that time. Over the observation period, eGFR_Cr_ remained relatively stable and linear, with an adjusted mean eGFR_Cr_ increase of +1.0 ml/min/1.73m^2^ for each 1-year increase in age according to our multivariable model (95%CI: 0.7 to 1.4; P<0.0001). This contrasted the significant decrease of -3.3 ml/min/1.73m^2^ per year of age observed in those <18 years of age using eGFR_CysC_ under the same conditions (95%CI: -4.2 to -2.4; P<0.0001), which then stabilized from the ages of 18 to 25 years (β=0.1; 95%CI: -0.1 to 0.2; P = 0.433). Using the combined eGFR_Cr-CysC_, a statistically non-significant decrease of -0.1 ml/min/1.73m^2^ was observed under adjusted conditions (95%CI: -0.4 to 0.2; P = 0.452). Similar trends in both direction and magnitude were observed when analyzed using diabetes duration as the time variable.

### 3.3 Longitudinal associations with eGFR; clinical measures & deprivation

Significant associations were observed between the combined eGFR_Cr-CysC_ and sex, insulin therapy, BMI, HbA1c and material deprivation quintile groups over time according to our final multivariable model (**Table 2**).

**Table 2:** Multivariable linear mixed effects outputs for eGFR_Cr_, eGFR_CysC_, eGFR_Cr-CysC_ & uACR.

Predictors	eGFR_Cr_			eGFR_CysC_			eGFR_Cr-CysC_			uACR		
	β	95%CI	p	β	95%CI	p	β	95%CI	p	β	95%CI	p
Intercept	91.2	77.9 – 104.4	<0.0001	154.2	137.3 – 171.1	<0.0001	112.5	102.0 – 123.0	<0.0001	2.04	0.42 – 3.65	0.014
Age, years	1.0	0.7 – 1.4	<0.0001	–	–	–	-0.1	-0.4 – 0.2	0.452	-0.03	-0.08 – 0.01	0.153
Age (0-18), years	–	–	–	-3.3	-4.2 – -2.4	<0.0001	–	–	–	–	–	–
Age (18-25), years	–	–	–	0.1	-0.1 – 0.2	0.459	–	–	–	–	–	–
Sex												
*Female [reference]*	–	–	–	–	–	–	–	–	–	–	–	–
*Male*	9.9	5.6 – 14.2	<0.0001	4.0	0.7 – 7.3	0.019	6.6	3.4 – 9.8	<0.001	-0.31	-0.74 – 0.12	0.159
Diabetes Duration (Baseline), years	-0.8	-1.4 – -0.1	0.027	-0.4	-0.9 – 0.1	0.128	-0.6	-1.1 – -0.1	0.030	-0.03	-0.10 – 0.03	0.317
Body Mass Index, kg/m^2^	-0.7	-1.0 – -0.3	0.006	-0.5	-0.8 – -0.2	0.002	-0.7	-1.0 – -0.4	<0.0001	-0.01	-0.05 – 0.03	0.729
Insulin therapy												
*Injections [reference]*	–	–	–	–	–	–	–	–	–	–	–	–
*CSII*	-4.5	-7.8 – -1.2	0.007	-2.0	-5.1 – 1.1	0.197	3.9	1.4 – 6.4	0.003	0.18	-0.57 – 0.20	0.349
HbA1c, mmol/mol	27.3	16.4 – 38.3	<0.0001	11.0	-1.1 – 21.9	0.062	14.2	5.5 – 23.0	0.001	0.4	0.9 – 1.9	0.491
HbA1c, %	2.5	1.5 – 3.5	<0.0001	1.0	-0.1 – 2.0	0.062	1.3	0.5 – 2.1	0.001	0.04	-0.08 – 0.17	0.491
Ethnic background												
*White [reference]*	–	–	–	–	–	–	–	–	–	–	–	–
*South Asian*	-0.6	-8.0 – 6.9	0.881	2.2	-3.6 – 8.1	0.453	1.0	-4.6 – 6.6	0.722	-0.02	-0.77 – 0.74	0.966
*Black*	-2.6	-12.2 – 7.1	0.600	5.8	-1.7 – 13.2	0.132	3.2	-4.0 – 10.5	0.377	-0.39	-1.35 – 0.57	0.425
*Chinese*	0.05	-9.3 – 9.4	0.992	5.3	-2.0 – 12.7	0.155	2.8	-4.3 – 9.9	0.430	-0.17	-1.10 – 0.75	0.711
*First Nations*	2.5	-22.7 – 27.6	0.847	6.4	-12.4 – 25.2	0.501	4.0	-14.7 – 22.7	0.676	0.47	-1.99 – 2.93	0.709
*Other*	-2.0	-7.7 – 3.7	0.498	2.7	-1.8 – 7.3	0.238	-0.03	-4.3 – 4.3	0.991	0.36	-0.22 – 0.94	0.221
MD Quintile groups												
*Q1-Q3 (High SES) [reference]*	–	–	–	–	–	–	–	–	–	–	–	–
*Q4-Q5 (Low SES)*	9.3	3.8 – 14.9	0.0012	3.1	-1.2 – 7.4	0.152	6.5	2.3 – 10.6	0.0025	0.38	-0.17 – 0.93	0.171

Differences in eGFR were observed between sexes over time using the combined eGFR_Cr-CysC_, with males having an eGFR_Cr-CysC_ +6.6 ml/min/1.73m^2^ higher than females over adolescence and early adulthood (95%CI: 3.4 to 9.8; P<0.001). When categorized using a cutoff of 135ml/min/1.73m^2^, males were found to have 4.4 times the odds of being flagged for hyperfiltration when compared to females using eGFR_Cr-CysC_ (95%CI: 1.4 to 10.3; P = 0.011). Inversely, females had 5.3 times the odds of being flagged with eGFR values <90ml/min/1.73m^2^ using eGFR_Cr-CysC_ under the same controlled conditions (95%CI: 2.3 to 11.9; P<0.0001).

Significant differences in eGFR were also observed between insulin therapy groups, with individuals on injection therapy having an eGFR_Cr-CysC_ +3.9 ml/min/1.73m^2^ greater than those on CSII therapy over time (95%CI: 1.1 to 6.4; P = 0.0025). For BMI, a lower eGFR_Cr-CysC_ of -0.6 ml/min/1.73m^2^ was observed for each 1 kg/m^2^ elevation in body mass (95%CI: -0.9 to -0.4; P<0.0001). For HbA1c, eGFR_Cr-CysC_ increased by +1.3 ml/min/1.73m^2^ for each 11 mmol/mol (1.0%) increase in HbA1c (95%CI: 5.5 to 23.0; P = 0.0014). Of these variables, only HbA1c was found to be associated with hyperfiltration above 135 ml/min/1.73m^2^, with the odds of HF increasing by 1.7 times for each 11 mmol/mol (1.0%) increase in HbA1c under adjusted conditions (95%CI: 12.0 to 23.0; P = 0.029).

A considerable difference in eGFR was observed between individuals from the least deprived MD quintiles (Q1-Q3) when compared to those from the most deprived MD quintiles (Q4-Q5). According to our model, mean eGFR_Cr-CysC_ was +6.5 ml/min/1.73m^2^ greater among those from the Q4-Q5 MD quintiles than those from the Q1-Q3 over time (95%CI: 2.3 to 10.6; P = 0.0025). This trend was largely driven by eGFR_Cr_ (Δ: +9.3; 95%CI: 3.8 to 14.9; P = 0.0012) rather than eGFR_CysC_ (Δ: +3.4; 95%CI: -0.8 to 7.6; P = 0.108). When analyzed in relation to hyperfiltration, a significant difference in the odds of HF was observed between MD quintiles under unadjusted conditions. In a simple model adjusting only for time (age), individuals from the Q4-Q5 quintiles had 3.0 times the odds of HF when compared to those in the Q1-Q3 quintiles (P = 0.027). This difference, however, decreased in magnitude and became borderline significant once adjusted (OR: 2.6; P = 0.054). Similar trends were observed when defining hyperfiltration using the threshold of 126.8ml/min/1.73m^2^ derived from the National Health and Nutrition Examination Survey (NHANES) data (30).

### 3.4 Longitudinal trends in uACR and associations with clinical and social factors

A total of N = 646 uACR data points derived from early morning urine samples collected alongside eGFR were used in this analysis. Baseline uACR for the overall sample was 1.43 ± 1.88 mg/mol. Under uncontrolled conditions, a mild but significant decrease in mean uACR of –0.04 mg/mol per year was observed over time from ages 10 to <25 years (95%CI: -0.08 to -0.003; P = 0.036) (See **Supplementary Figure 2**). Once covariates were added to the model, the slope in uACR over time (age) could not be differentiated from zero (β: -0.032; 95%CI: -0.08 to 0.12; P = 0.153). In contrast to eGFR, none of the variables added as fixed effects were found to be associated with raw uACR in mg/mol over time as shown in **Table 2**. However, significant longitudinal associations were observed with sex (P = 0.017), baseline diabetes duration (P = 0.049) and HbA1c (P = 0.021) were observed once uACR was log-transformed. When categorized for moderately elevated albuminuria using a cutoff of 3mg/mol, only age (P = 0.027) and ethnicity were associated over time. No associations with predictors were observed when models were re-run using the Diabetes Canada-recommended cutoff of ≥2 mg/mol to define moderately elevated albuminuria (31).

## 4 Discussion

The current study evaluated eGFR trends over a 10-year period across adolescence into early adulthood in youth with type 1 diabetes and assessed longitudinal associations between eGFR and a combination of clinical and social factors. eGFR was stable across adolescence into early adulthood in youth with type 1 diabetes using the combined age- and sex-dependent creatinine and cystatin c eGFR formula when controlling for factors such as sex, BMI, baseline diabetes duration, insulin therapy, HbA1c, ethnicity and SES strata.

While significant increases and decreases in eGFR were observed using creatinine-derived eGFR and cystatin c-derived eGFR, respectively, these trends were generally mild and were eliminated with the use of the combined CKiD U25_Cr-CysC_ eGFR. A large, clinically relevant decline in eGFR (>3 ml/min/1.73m^2^) was observed from ages 10 to <18 years using the CKiD U25_CysC_ formula, which stabilized thereafter from ages 18 to 25 years. In adults, a more rapid eGFR decline has been associated with progression to renal disease. However, the immediate stabilization in eGFR at age 18 combined with low rates of moderate elevations in albumin (ACR >3mg/mol) does not suggest maladaptive GFR decline in the overall cohort during adolescence. Instead, this trend may be driven by the design of the age- and sex-adjusted CKiD U25 formula, which uses a constant for individuals above 18 years of age when compared to the age-dependent K values used for those below this age threshold.

**Figure 1:**
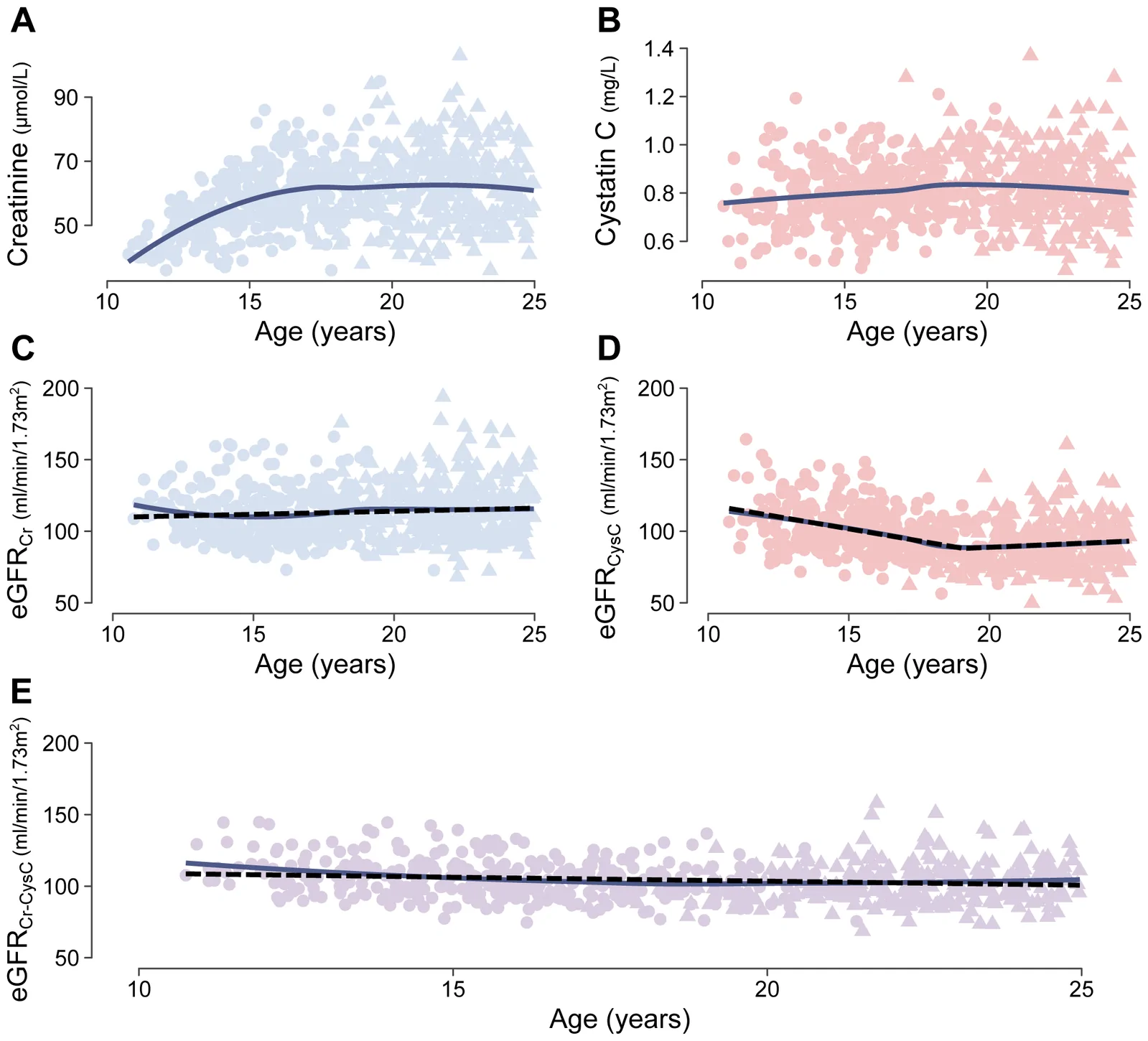
Scatterplots of **(A)** creatinine in μmol/L, **(B)** cystatin c in mg/L, **(C)** CKiD U25_Cr_ eGFR in ml/min/1.73m^2^, **(D)** CKiD U25_CysC_ eGFR in ml/min/1.73m^2^ & **(E)** CKiD U25_Cr-CysC_ eGFR in ml/min/1.73m^2^ by age in years. Data points collected as part of AdDIT are presented as circles (●) while those collected as part of the CanSOLVE CKD-SPOR follow-up study are presented as triangles (▲). LOESS regression lines displaying smoothed eGFR trends over time are presented as solid dark blue lines (**–**) while unadjusted linear regression slopes used to approximate trends in eGFR over time are presented as dashed black lines (**– –**).

According to the original Pierce et al. (26) paper, the largest corrections in K values for age are from 12 to 18 year of age as these coincide with traditional ages of pubertal growth. The corrections are made to account for varying levels of serum creatinine and cystatin c produced as the body grows, as illustrated by the changes in creatinine shown in **Figure 1A**. However, as cystatin c levels remained fairly stable across the age range in our cohort (see **Figure 1B**), the application of this correction resulted in a negative slope across the adolescent age range when using the CKiD U25_CysC_ formula. While this was ultimately corrected by combining the CKiD U25_Cr_ and CKiD U25_CysC_ formulas, our data suggests the sole application of the CKiD U25_CysC_ formula in adolescents with type 1 diabetes may lead to an overestimation of “rapid decliners” despite the absence of other signs of DKD progression. When using either the CKiD U25_Cr_ and CKiD U25_Cr-CysC_ formulas, we observed that eGFR was generally stable across the 10 to 25 year age range, which is similar to trends from previous reports that demonstrated direct measures of GFR using was relatively stable during the transition from pediatric to adult care (6).

The evaluation of traditional clinical factors such as sex, BMI, insulin therapy and HbA1c, and non-traditional risk factors including material deprivation were found to be significantly associated with eGFR. Our study highlighted negative associations between eGFR and markers of glycemic management which are intriguing and suggestive that better management practices such as CSII therapy and lower HbA1c attenuate eGFR over the course of adolescence and early adulthood. These findings align with literature describing reductions in oxidative stress and advanced glycation end products (AGEs) associated with stricter glycemic management that is achieved with insulin pump therapy (32).

Clinically meaningful differences in eGFR were also observed between sexes and deprivation groups. More specifically, males were found to have significantly higher eGFR values and higher odds of hyperfiltration relative to females across the age range of 10 and 25 years. Further evaluation the eGFR slopes for males and females confirmed these were parallel over time but consistently higher in males across the age range. While there is mixed evidence of sex as a risk factor for the development of DKD in adults with diabetes (33, 34), one recent study in youth with type 1 diabetes found an increased risk of rapid eGFR decline (<−3 ml/min/1.73m^2^ per year) among females relative to males under 18 years of age using the same age- and sex-dependent CKiD U25 Cr-based eGFR formula used in our study (9). This contrasts with another study in adolescents with type 1 diabetes which observed a greater incidence of hyperfiltration among adolescent females when compared to age-matched males (35). The difference between our findings may be related to their use of the Larsson eGFR formula. As Larsson eGFR does not adjust for sex, this would inherently increase the overall estimates of GFR for females relative to males when compared to the CKiD U25 formulas, which automatically assign lower K values for females by design. The result of the adjustment for sex in the CKiD U25 appears to drive the significant differences in eGFR observed according to sex in our models rather than true underlying differences in GFR between sexes as further evaluations of mGFR in a subset of n=53 participants from this cohort confirmed no differences in mGFR were observed between sexes (36). This ultimately suggests that the sex-based differences in eGFR observed in our study may be driven by mathematical or formulaic factors rather than true differences in kidney function between sexes. The repercussions of this are clinically relevant given that, according to our data, the overestimation of the difference in GFR between sexes may lead to higher rates of false positivity among males for hyperfiltration regardless of the threshold used given that there is no standard definition for hyperfiltration in youth with T1D. The opposite would be true for females, whereby greater rates of false positives for eGFR values <90 ml/min/1.73m^2^ would be expected despite similar underlying GFR values. According to clinical practice guidelines, this would prompt additional testing that may not be necessary (1).

Significant differences in eGFR were also observed between individuals from high vs. low socioeconomic strata. The parallel slopes observed in our models suggest this difference in GFR persists from adolescence to early adulthood. While DKD is associated with decreased eGFR, supraphysiologic increases in GFR linked to intermittent hyperfiltration may be indicative of underlying kidney dysfunction. Recent evidence regarding the classification of longitudinal eGFR trajectories in adults with type 1 diabetes highlights that elevated eGFR levels in the early years following diabetes diagnosis are at greater risk for “steep decline” during adulthood (10). While social determinants were not directly evaluated as part of the report by Favel et al., factors strongly associated with SES including HbA1c and BMI were highlighted as potential risk factors for steeply declining eGFR trajectories. Beyond glycemia and anthropometrics, additional factors related to SES that may explain the differences in kidney function observed in our study include dietary intake, diabetes technology use (ex. use of CGM, insulin pump therapy, etc.) and interactions with the healthcare system (37–39). The cumulative effect of these factors helps describe early alterations in renal function that over time may contribute to the higher rates of DKD observed in individuals with type 1 diabetes from lower SES backgrounds (40).

This study includes numerous strengths including its well characterized cohort of youth with type 1 diabetes with Cr and CysC measurements collected at the same time points, which allowed the evaluation of combined eGFR shown to be more accurate than the individual eGFRs derived from each marker (28). This study followed adolescents with type 1 diabetes for more than 10 years of GFR data during a critical phase of CKD risk development as youth transitioned from pediatric to adult care. Nonetheless, additional longitudinal studies on this topic with larger samples sizes are warranted to confirm and validate the findings presented in this study. The inclusion of traditional and non-traditional risk factors in our analysis allowed for a more comprehensive evaluation of GFR in the context of type 1 diabetes, highlighting the important role played by social factors linked to SES that are often excluded from clinical studies. This study also included limitations and should be interpreted accordingly. The cohort from this *post-hoc* study was evaluated over the course of two studies for which the longitudinal evaluation of GFR over time was not a pre-established objective. In addition, the use of a single eGFR formula for creatinine and cystatin c may also be considered a limitation and different trends in eGFR may have been observed using a different set of eGFR formulas. The selection of the CKiD U25 formulas was based on recommendations from clinical practice guidelines and on a prior assessment of eGFR formula performance in youth with type 1 diabetes which showed this set of equations performed well as approximating mGFR in this population (1, 36). However, applying this formula required excluding observations after age 25, resulting in data loss and fewer observations per individual. Another limitation pertains to the results surrounding ethnicity as these should be interpreted with caution as well given the small number of participants within several of the groups. Lastly, the reliance on estimated GFR rather than its gold standard in measured GFR for the evaluation of kidney function over time may also be considered a limitation for this study.

In the absence of an eGFR equation validated for youth with type 1 diabetes, the results of this study are useful to better understand how the application of recommended eGFR formulas affects the assessment of kidney function in youth with type 1 diabetes over time. Our data provides clinically meaningful insights into factors impacting kidney function over time and emphasizes to clinicians that, while a single eGFR value is an estimate at single point in time may be helpful, evaluating eGFR trends are more informative to assess kidney health and changes in kidney function over time. In youth with diabetes with preserved kidney function (eGFR ≥90 mL/min/1.73 m^2^), those with persistent elevations in GFR consistent with hyperfiltration or rapid declines in GFR may benefit from further optimization of glycemic management, urine ACR monitoring, hypertension screening and healthy weight management counseling. While research surrounding these targeted interventions are well documented in adults with type 1 diabetes, further studies in this area are needed in pediatrics to confirm their effectiveness in this subpopulation (41–44). Given updated ADA and ISPAD pediatric clinical practice guidelines now recommend eGFR testing, the findings from this study provide additional longitudinal evidence highlighting the clinical utility and caveats of the updated CKiD U25 equations when applied in youth with diabetes.

## Data Availability

The raw data supporting the conclusions of this article will be made available by the authors, without undue reservation.
