# Supplementary material for: Longitudinal evaluation of glomerular filtration rate across adolescence and early adulthood in youth with type 1 diabetes: associations with clinical and social risk factors
Source: Front Endocrinol (Lausanne). 2026 Aug 26;17:1888628. doi: 10.3389/fendo.2026.1888628 (PMC13553482; doi:10.3389/fendo.2026.1888628)
Supplement: Supplementary file 1 [file DataSheet1.docx]

## Supplementary Materials

**Supplementary Figure S1.** A) Mean baseline eGFR levels in ml/min/1.73m^2^ by Material Deprivation (MD) quintiles, and B) mean baseline eGFR levels in ml/min/1.73m^2^ by MD quintile groups. Q1 represents the least deprived quintile while Q5 encompasses the most deprived individuals. MD quintile groups were formed by grouping the 3 least deprived quintiles (i.e., Q1-Q3) and the 2 most deprived quintiles (Q4-Q5). Error bars represent 95% confidence intervals.

**Supplementary Figure S2.** A) Urine Albumin-Creatinine Ratio (uACR) in mg/mol and B) log uACR in log mg/mol over Age in years. Simple linear regression lines displaying raw ACR trends over time are represented as dotted black lines.

**Supplemental Table S1.** Baseline characteristics of study participants by MD16 quintile group.

| **Baseline Characteristics** | **Q1-Q3**  n = 106 | **Q4-Q5**  n = 31 | **p** |
| --- | --- | --- | --- |
| Age, years | 14.0 ± 1.6 | 13.3 ± 1.3 | 0.012 |
| Diabetes Duration, years | 6.1 ± 3.4 | 5.9 ± 2.9 | 0.729 |
| Sex |  |  |  |
| *Female*, n (%) | 51 (48.1) | 13 (41.9) | 0.544 |
| *Male*, n (%) | 55 (51.9) | 18 (58.1) |  |
| Body Mass Index, kg/m^2^ | 21.8 ± 3.9 | 22.2 ± 5.0 | 0.702 |
| SBP, mmHg | 113.7 ± 9.8 | 113.9 ± 10.8 | 0.936 |
| DBP, mmHg | 66.0 ± 7.4 | 68.3 ± 9.2 | 0.209 |
| Insulin Therapy |  |  | 0.209 |
| *CSII*, n (%) | 69 (65.1) | 16 (51.6) |  |
| *Injections*, n (%) | 37 (34.9) | 15 (48.4) |  |
| HbA1c, mmol/mol | 65 ± 11 | 62 ± 13 | 0.500 |
| HbA1c, % | 8.1 ± 1.0 | 7.8 ± 1.2 | 0.500 |
| Ethnic Background |  |  |  |
| *White*, n (%) | 72 (67.9) | 7 (22.6) | <0.0001 |
| *South Asian*, n (%) | 7 (6.6) | 6 (19.4) |  |
| *Black*, n (%) | 6 (5.7) | 2 (6.5) |  |
| *Chinese*, n (%) | 6 (5.7) | 2 (6.5) |  |
| *First Nations*, n (%) | 1 (0.9) | 0 (0.0) |  |
| *Other*, n (%) | 14 (13.2) | 14 (45.2) |  |
| eGFR_Cr_ (CKiD U25), ml/min/1.73m^2^ | 108.9 ± 15.9 | 118.4 ± 15.2 | 0.004 |
| eGFR_CysC_ (CKiD U25), ml/min/1.73m^2^ | 107.1 ± 17.6 | 114.4 ± 21.9 | 0.096 |
| eGFR_Cr-CysC_ (CKiD U25), ml/min/1.73m^2^ | 108.0 ± 13.5 | 116.4 ± 15.7 | 0.01 |
| uACR, mg/mmol | 1.51 ± 1.82 | 1.13 ± 2.06 | 0.365 |
| Albuminuria (moderately elevated) |  |  |  |
| *<3 mg/mol*, n (%) | 91 (85.8) | 30 (96.8) | 0.120 |
| *≥3 mg/mol*, n (%) | 15 (14.2) | 1 (3.2) |  |
